# Route planning of mobile robot based on improved RRT star and TEB algorithm

**DOI:** 10.1038/s41598-024-59413-9

**Published:** 2024-04-18

**Authors:** Xiong Yin, Wentao Dong, Xiaoming Wang, Yongxiang Yu, Daojin Yao

**Affiliations:** School of Electrical and Automation Engineering, East China Jiaotong University, Nanchang, 330000 China

**Keywords:** RRT* algorithm, TEB algorithm, Path planning, AGV, Kinematic, Electrical and electronic engineering, Mechanical engineering

## Abstract

This paper presents a fusion algorithm based on the enhanced RRT* TEB algorithm. The enhanced RRT* algorithm is utilized for generating an optimal global path. Firstly, proposing an adaptive sampling function and extending node bias to accelerate global path generation and mitigate local optimality. Secondly, eliminating path redundancy to minimize path length. Thirdly, imposing constraints on the turning angle of the path to enhance path smoothness. Conducting kinematic modeling of the mobile robot and optimizing the TEB algorithm to align the trajectory with the mobile robot's kinematics. The integration of these two algorithms culminates in the development of a fusion algorithm. Simulation and experimental results demonstrate that, in contrast to the traditional RRT* algorithm, the enhanced RRT* algorithm achieves a 5.8% reduction in path length and a 62.5% decrease in the number of turning points. Utilizing the fusion algorithm for path planning, the mobile robot generates a superior, seamlessly smooth global path, adept at circumventing obstacles. Furthermore, the local trajectory meticulously conforms to the kinematic constraints of the mobile robot.

## Introduction

With the rapid advancements in robot technology, the potential for robots to supplant human roles has emerged^1,2^. The mobile robot stands out prominently in both domestic and international arenas as one of the focal points in robot research^3^. Its applications span across hospitals, railway stations, warehouses, and shopping malls^4^. With robots capable of operating continuously, a foreseeable shift involves them taking over repetitive manual tasks from humans^5^. At the core of this shift lies the essence of path planning for mobile robots^6^.

Currently, a multitude of algorithms cater to mobile robot path planning. These encompass the Voronoi algorithm^7^, A^*^ algorithm^8^, ant colony algorithm^9^, grey Wolf algorithm^10^, genetic algorithm^11^, RRT algorithm^12^, particle swarm algorithm^13^, ant colony algorithm^14^, artificial potential field algorithm^15^, neural network algorithm^16^, deep learning algorithm^17^, and more.

Path planning algorithms can be categorized into global path planning and local path planning based on their characteristics^18^. Among global planning algorithms, the RRT* algorithm, which employs sampling for global path planning, is widely adopted in mobile robot applications due to its accelerated path generation rate. Numerous researchers have contributed enhancements to the RRT* algorithm, including the bidirectional RRT* algorithm^19^, Informed RRT* algorithm^20^, and others.

In the realm of path planning, numerous algorithms have been proposed by scholars^21,22^. Tang et al. ^23^ introduces an enhanced A* algorithm within the domain of global path planning. The method consists of two main steps: firstly, removing irregular waypoints with functions P (x, y) and W (x, y); secondly, smoothing the path using B splines. This approach significantly reduces both the path length and the number of nodes. Meanwhile, Mashayekhi et al.^24^ delve into a hybrid RRT algorithm. Their method begins by employing a bidirectional tree for searching, then optimizing the current node through informed sampling once the two trees are connected. This technique notably enhances the path quality. Moving on, Zhu et al.^25^ adopt a reverse labeling Dijkstra algorithm for global path planning. Initially, they theoretically substantiate the algorithm's rationality and demonstrate its low complexity. Subsequently, they validate its effectiveness by applying it to a real road network. In the realm of local path planning, Zhang et al.^26^ present an improved ant colony algorithm. The strategy includes using the artificial potential field to determine force direction in the initial ship phase, improving the attraction potential field function, and creating pseudo-random transfer rules. These changes significantly improve the algorithm's convergence. Similarly, Du et al.^27^ propose a dynamic artificial potential field algorithm. The strategy begins by adjusting the safe distance dynamically and then improving the potential field force's effectiveness. Ultimately, the algorithm is enhanced by introducing a steering force to change the drone's direction, ensuring safer and more stable paths. Lastly, Kobayashi et al.^28^ introduce an algorithm comprising the DWA algorithm and VM. This composite approach furnishes the DWA algorithm with modified variable speeds and predicted obstacle data from VM, facilitating the generation of candidate paths.

While the mentioned algorithms have their merits, they mainly concentrate on either global or local path planning, frequently overlooking the robot's kinematic and dynamic constraints. This paper addresses this limitation by incorporating considerations for both global and local path planning, as well as accounting for the kinematics of the mobile robot. The study includes simulating the improved algorithm, comparing it with classical algorithms. Then implementing it on an experimental prototype to confirm its effectiveness.

In summary, propose a fusion algorithm for a mobile robot to navigate along an optimal path globally while obeying its kinematic constraints. This contribution differs from existing work in three key aspects:

1. The paper introduces the mobile robot platform and its system, followed by kinematic modeling for the mobile robot;
2. Enhancements to the RRT* algorithm include optimizing the sampling function. During node expansion, weights are increased to counteract expansion. Redundant path nodes are eliminated to shorten the path. Constraints are applied to turning angles for a smoother trajectory;
3. Addressing the kinematic constraints of the mobile robot, the TEB algorithm is optimized, incorporating constraints such as obstacle limitations, speed restrictions and acceleration constraints;
4. The effects of Angle threshold and path-point spacing on the performance of improved RRT* algorithm are discussed.

This paper is structured into six sections, with "Introduction of mobile robot" detailing the robot modeling, "Improved RRT* Algorithm" focusing on RRT* algorithm improvements, "Timed-elastic-band approach optimization" concentrating on TEB algorithm optimization, and "Experimental" encompassing simulations and experiments. Finally, "Conclusion" provides the conclusion of this study.

## Introduction of mobile robot

### Platform hardware introduction

**Figure 1:**
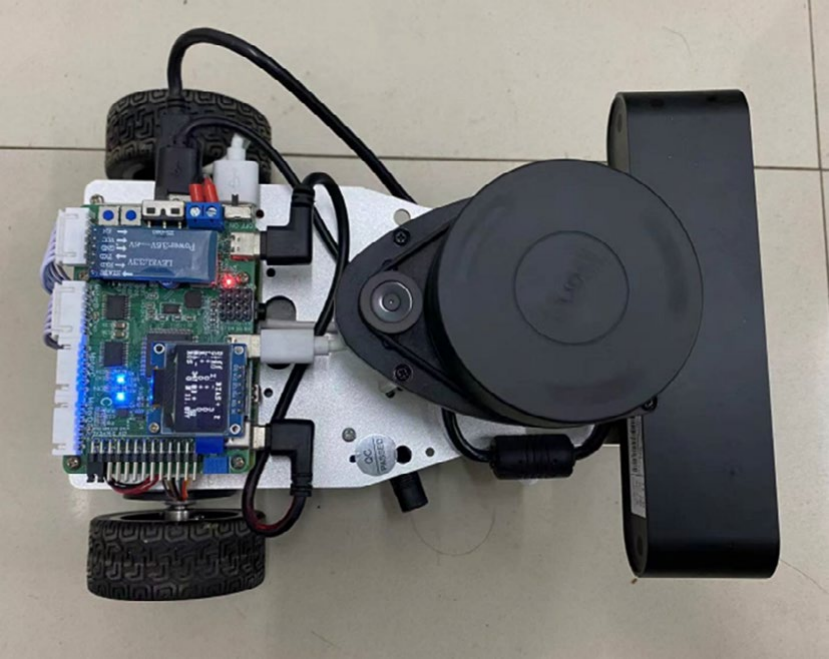
Mobile robot diagram.

The mobile robot comprises a depth camera, laser radar, main control unit and robot chassis. The main control unit is built around a Raspberry Pi, incorporating an STM32 chip. The robot chassis is constructed with two servo motors, two driving wheels, and an omnidirectional wheel, as depicted in Fig. 1.

### Platform system introduction

**Figure 2:**
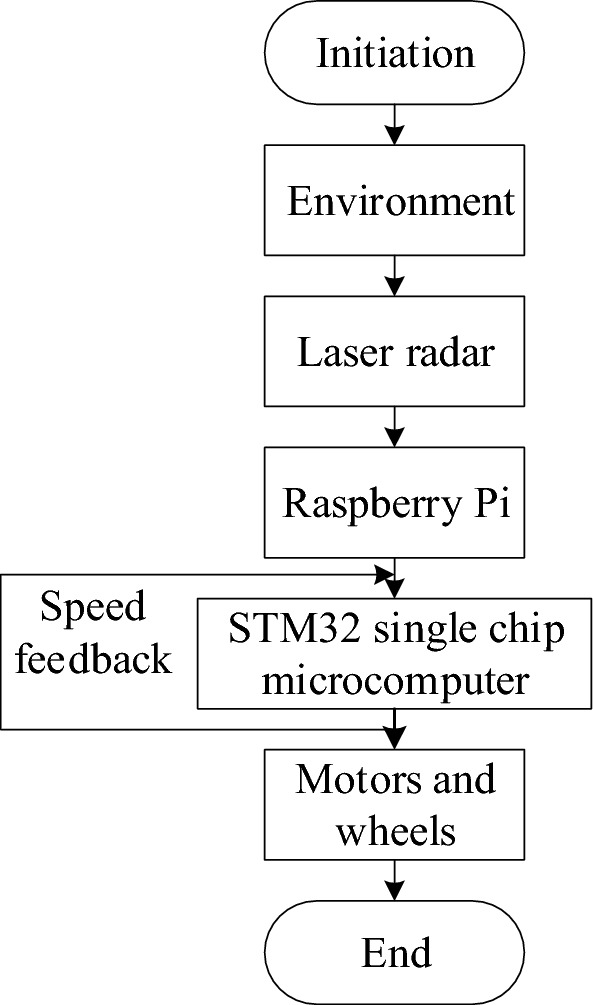
System flow chart.

The platform system integrates the ROS operating system into Raspberry Pi, with key components encompassing environment awareness, data processing and path navigation. ROS, widely recognized as the most extensively used open-source robot software platform, greatly improves the efficiency of robot development. The laser radar collects environmental data, while the Raspberry Pi and STM32 chip work together to process it and plan an optimal, obstacle-free path. The servo motor is controlled to start tire rotation, helping the mobile robot move along the planned path, as illustrated in Fig. 2.

### Kinematic model of the mobile robot

**Figure 3:**
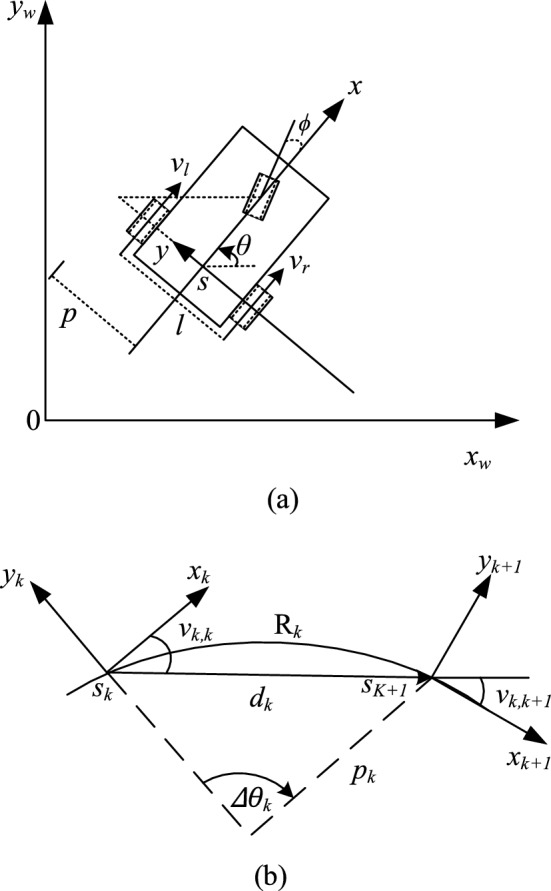
Mobile robot shape and posture diagram.

This paper conducts kinematic modeling for the mobile robot^29^. Within the world coordinate system (*x*_*w*_, *y*_*w*_), the state vector *s* = [*x*, *y*, *θ*] denotes the current position and pose information of the mobile robot. This is depicted in Fig. 3.

Given that the mobile robot operates on a two-wheeled differential system, each drive wheel is independently powered by a dedicated motor. Here, *r* represents the radius of the driving wheel, while *w*_*l*_(*t*) and *w*_*r*_(*t*) denote the angular speed of the left and right driving wheels, respectively. The linear speed of the left and right drive wheels is expressed as *v*_*l*_(*t*) and *v*_*r*_(*t*).

1 $$ \begin{gathered} v_{l} (t) = w_{l} (t)r \hfill \\ v_{r} (t) = w_{r} (t)r \hfill \\ \end{gathered} $$

where *t* represents the turning time period, *p* is half of the wheelbase of the driving wheel. Consequently, the local coordinate system for the mobile robot positions the *y*-axis along the horizontal direction of its two driving wheels, with the *x*-axis aligned with its moving direction. The speed of the mobile robot, denoted as *v*(*t*). Speed of the mobile robot *v*(*t*) = (*v*_*l*_(*t*) + *v*_*r*_(*t*))*/2*. This leads to the conclusion that the motion of the mobile robot can be characterized by a nonlinear equation.

2 $$ \dot{S}(t) = \left[ {\begin{array}{*{20}c} {\dot{x}(t)} \\ {\dot{y}(t)} \\ {\dot{\theta} (t)} \\ \end{array} } \right] = \left[ {\begin{array}{*{20}c} {v(t)\cos (\theta (t))} \\ {v(t)\sin (\theta (t))} \\ {w(t)} \\ \end{array} } \right] $$

where *s*(*t*) represents the position of the mobile robot at time *t*, *u*(*t*) = [*v*(*t*), *w*(*t*)]^T^ denotes the control input vector.

3 $$ u(t) = \left[ {\begin{array}{*{20}c} {v(t)} \\ {w(t)} \\ \end{array} } \right]{ = }\left[ {\begin{array}{*{20}c} {\frac{{v_{l} (t) + v_{r} (t)}}{2}} \\ {\frac{{v_{l} (t) - v_{r} (t)}}{l}} \\ \end{array} } \right] $$

where *l* is two driving wheels wheelbase, *v*_*l*_(t) is the left-wheel speed, *v*_*r*_(t) is the right-wheel speed, *v*_*k,k*_ is the angle between *s*_*k*_ pose at moment *k*, the direction *d*_*k*_ = [*x*_*k*+*1*_-*x*_*k*_, *y*_*k*+*1*_-*y*_*k*_, 0]^T^ of the mobile robot. *v*_*k,k*+*1*_ is the angle between the *d*_*k*_ direction and *s*_*k*+*1*_ posture of the mobile robot at time *k* + *1*.When *v*_*k,k*_ = *v*_*k,k* +*1*_, a common arc of constant curvature can be obtained.

4 $$ v_{k,k} = v_{k,k + 1} $$

5 $$ h_{k} (s_{k + 1} ,s_{k} ) = \left( {\left[ {\begin{array}{*{20}c} {\cos (\theta_{k} )} \\ {\sin (\theta_{k} )} \\ 0 \\ \end{array} } \right] + \left[ {\begin{array}{*{20}c} {\cos (\theta_{k + 1} )} \\ {\sin (\theta_{k + 1} )} \\ 0 \\ \end{array} } \right]} \right) \times d_{k} = 0 $$

According to Fig. 3b, *p*_*k*_ is the turning radius, arc length *R*_*k*_ = *p*_*k*_*∆θ*_*k*_. Angle *∆θ*_*k*_ = *θ*_*k*+*1*_ − *θ*_*k*_ is the angle change between the *s*_*k*_ position and *s*_*k*+*1*_ position of the mobile robot. The turning radius *p*_*k*_ is.

6 $$ p_{k} = \frac{{\left\| {d_{k} } \right\|_{2} }}{{\left| {2\sin (\frac{{\vartriangle \theta_{k} }}{2})} \right|}}\mathop \approx \limits^{{\vartriangle \theta_{k} \ll 1}} \frac{{\left\| {d_{k} } \right\|_{2} }}{{\left| {\vartriangle \theta_{k} } \right|}} $$

where meet *p*_*k*_ ≥ *p*_*min*_, *p*_*min*_ is the minimum turning radius.

## Improved RRT* algorithm

### Traditional RRT* algorithm

The Rapidly Exploring Random Tree Star (RRT*) stands as a global path planning algorithm, representing an enhancement over the Rapidly Exploring Random Tree (RRT)^30^. The core method involves several steps: first, randomly sampling within the space. Once a sample point is obtained, the algorithm finds the nearby node. Second, a new node is created between this nearby node and the sampled one. Next, collision detection is done on the new node. If there's no collision, it's added to the path. The parent node is then adjusted for the new one. This continues until the algorithm reaches the target point.

### Adaptive sampling function

In the conventional RRT* algorithm, the initial path is formed through random sampling across the entire obstacle space, resulting in a considerable number of sampling nodes. To enhance the target-oriented nature of sampling points, an adaptive sampling function is introduced. A proposed adaptive sampling function involves setting a target bias probability denoted as *p*. Generate sampling points based on the associated probability. This adaptive sampling function accelerates the generation of the initial path, reduces the quantity of sampling points, improves sampling efficiency, and prevents the initial path from succumbing to local optima. The adaptive sampling function is instrumental in achieving these enhancements.

7 $$ X_{rand} = \left\{ {\begin{array}{*{20}c} {x_{rand} (p = 0.8)} \\ {X_{Goal} (p = 0.2)} \\ \end{array} } \right., \quad p \in (0,1) $$

where *p* represents probability, *x*_*rand*_ denotes the sampling point generated by random sampling, *X*_*Goal*_ signifies target point, *X*_*Goal*_ is sampling point.

### Node bias expansion

**Figure 4:**
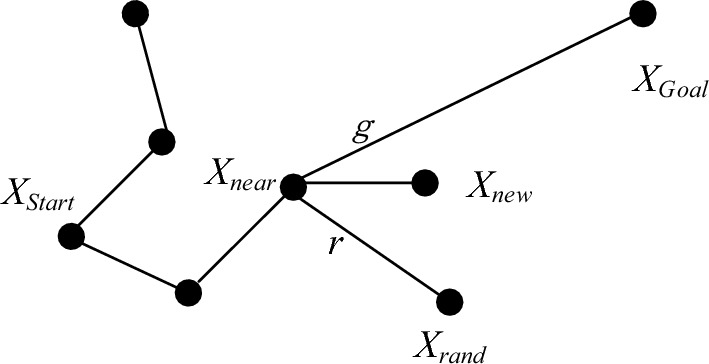
New node offset expansion diagram.

Given the relatively lengthy trajectory of the mobile robot, it is essential to constrain the expansion direction of the new node, denoted as *X*_*new*_. This constraint is crucial for expediting path planning^31^. In the traditional RRT* algorithm, the new node *X*_*new*_ is traditionally extended towards a randomly generated point, *X*_*rand*_. However, this random expansion approach often results in an unfocused expansion of nodes, leading to prolonged path planning durations. To address this inefficiency, this paper introduces a node expansion bias. This bias leverages target points to guide and constrain the expansion of new nodes. Specifically, different weights are assigned to the direction of the target point and the sampling point. The weight in the direction of the goal point, *X*_*Goal*_, is denoted as *g*, while the weight in the *X*_*rand*_ direction is denoted as *r*. This configuration is illustrated in Fig. 4. The introduction of this node expansion bias aims to optimize the expansion process and enhance the efficiency of path planning.

### Remove redundant points in the path

**Figure 5:**
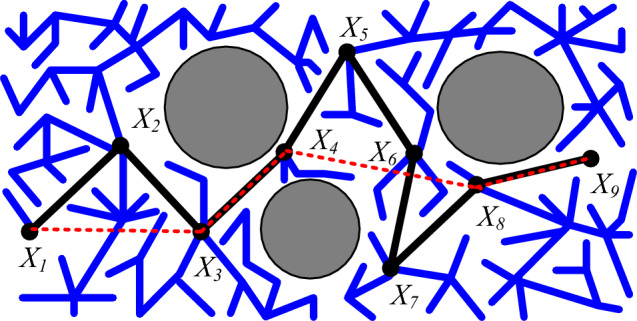
Path de-redundancy diagram.

The path generated from the starting point to the destination point often contains numerous redundant points, contributing to the elongation of the path^32^. Reducing redundant points decreases the time needed for the mobile robot to complete the task, making it essential to trim them for a collision-free optimal path. This paper employs the greedy algorithm for path redundancy reduction. In Fig. 5, the blue line segment represents the branch of the random tree, the black line segment corresponds to the initial path, the red dotted line indicates the path after redundancy removal, and the gray circles symbolize obstacles. To remove redundancy path: *X*_*1*_ $\to$ *X*_*2*_ $\to$ *X*_*3*_ $\to$ *X*_*4*_ $\to$ *X*_*5*_ $\to$ *X*_*6*_ $\to$ *X*_*7*_ $\to$ *X*_*8*_ $\to$ *X*_*9*_. Iterate through the path in turn. Until node *X*_*4*_ is encountered, connecting nodes *X*_*1*_ and *X*_*4*_ will collide with obstacles. Connect *X*_*1*_ and *X*_*3*_, delete node *X*_*2*_. Repeat the process until reaching node *X*_*9*_. The path with redundancy removed can be obtained: *X*_*1*_ $\to$ *X*_*3*_ $\to$ *X*_*4*_ $\to$ *X*_*8*_ $\to$ *X*_*9*_. As shown in the Fig. 5.

### Path smoothing

**Figure 6:**
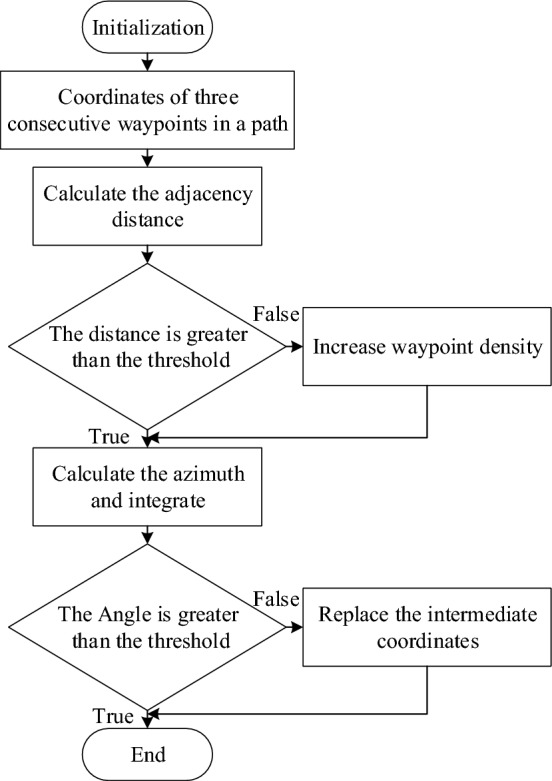
Path smoothing schematic diagram.

Once redundancy is eliminated, the path may still face issues with excessively wide turning angles. The path turning angle is too large, which does not conform to the kinematics of the mobile robot. Therefore, it is necessary to constrain the turning angle of the path. Firstly, the coordinates of three consecutive waypoints are obtained. The angular deviation of two adjacent coordinates is calculated. The angle deviation threshold *A*_*d*_ is set. The calculated result is greater than the threshold. Then, the midpoint of the preceding. Following two points is used as the coordinates of the intermediate points, so as to achieve path smoothing. Secondly, the angle normalization function is set up. It combines angles from two nearby points in the map system into the range of (−π, π) to make angle comparisons consistent. Obtain the coordinates of the current time and the previous time waypoint. Calculate the distance. If the distance obtained is greater than the threshold, a new waypoint is inserted between the two waypoints. The coordinate is the point coordinate of the two path points, so as to increase the path point density. Finally, the path smoothing function is established to obtain the coordinates of three adjacent waypoints. Then the angle value between two adjacent coordinates is calculated. The two angle standardization deviations are obtained by calculating and analyzing the angle standardization function. The calculation result is greater than the threshold. The middle coordinate points are substituted with the median value of the rear coordinate points. This process is repeated continuously to globally optimize the entire path, as shown in the Fig. 6.

## Timed-elastic-band approach optimization

### Basic description of algorithm

The Timed Elastic Band (TEB) algorithm is a popular method for tackling local obstacle avoidance problems faced by mobile robots^33^. This algorithm is formally defined as a tuple comprising two sequences:

8 $$ B: = (Q,\tau ) $$

where *Q* represents the sequence of robot poses, and *τ* signifies the sequence of time intervals associated with each pose. The basic idea is to dynamically adjust and optimize the algorithm with respect to configuration and time intervals. This is achieved through a real-time weighted multi-objective optimization process.

9 $$ f(B) = \sum\limits_{k} {\gamma_{k} f_{k} (B)} $$

10 $$ B* = \mathop {\arg \min }\limits_{B} f(B) $$

where B^*^ refers to the optimized Timed Elastic Band (TEB) algorithm, where *f*_*k*_ represents the weighted sum of components contributing to the objective function *f(B)*. The objective function consists of four integral elements: (1) A penalty function, strategically employed to guide the intermediate points toward the original path and circumvent obstacles. (2) Utilization of dynamics to impose restrictions on the robot's speed and acceleration. (3) Adherence to nonholonomic kinematic constraints. (4) Minimization of the square of the sum of the time difference sequence, aiming to identify the swiftest path. The TEB algorithm introduces a constraint on the robot's movement time, thereby enabling real-time control over the mobile robot's motion^34^. This additional constraint enhances the algorithm's ability to govern the movement of the mobile robot in dynamic environments^35^.

### Algorithm optimization

The application of the TEB algorithm involves optimizing both the dynamics and kinematics of a mobile robot^36^. The following is the optimization formula of TEB algorithm^37,38^. The main aim is to guide the robot smoothly from start to finish along the best path, considering dynamics and kinematics. The optimization problem is defined as a finite dimensional parameter vector (*s*_*k*_) _*k*=1,2,…,*n*−1_.consisting of a discretized sequence of *n* robot poses… *n* minus one. The parameter set to be optimized is:

11 $$ B: = \left\{ {s_{1} ,\Delta T_{1} ,s_{2} ,\Delta T_{2} , \ldots ,s_{n - 1} ,\Delta T_{n - 1} ,s_{n} } \right\} $$

where (∆*T*_*k*_) _*k*=*1,2,…,n−1*_ indicates strictly positive time series. Simultaneously, the optimization problem undergoes a transformation into a non-linear program, which involves a series of equations and inequalities.

12 $$ \mathop {\min }\limits_{B} \sum\limits_{k = 1}^{n - 1} {\Delta T_{k}^{2} } $$

Subject to

13 $$ \begin{array}{*{20}c} {s_{1} = s_{c} ,} & {s_{n} = s_{f} ,} & {0 \le \Delta T_{k} \le } \\ \end{array} \Delta T_{\max } , $$

14 $$ \begin{array}{*{20}c} {h_{k} (s_{k + 1} ,s_{k} ) = 0,} & r \\ \end{array}_{k} (s_{k + 1} ,s_{k} ) \ge 0, $$

15 $$ o_{k} (s_{k} ) \ge 0, $$

16 $$ \begin{array}{*{20}c} {v_{k} (s_{k + 1} ,s_{k} ,\Delta T_{k} ) \ge 0,} & {(k = 1,2, \ldots ,n - 1)} \\ \end{array} $$

17 $$ \alpha_{k} (s_{k + 2} ,s_{k + 1} ,s_{k} ,\Delta T_{k + 1} ,\Delta T_{k} ) \ge 0,(k = 2,3, \ldots ,n - 2) $$

18 $$ \alpha_{1} (s_{2} ,s_{1} ,\Delta T_{k + 1} ) \ge 0,\alpha_{n} (s_{n} ,s_{n - 1} ,\Delta T_{n - 1} ) \ge 0. $$

Among them, the initial pose *s*_*1*_ and the end pose *s*_*n*_ are replaced by the current mobile robot state *s*_*c*_ and target state *s*_*f*_. Equality constraint *h*_*k*_(∙)satisfies Eq. (5). *r*_*k*_(∙)imposes a minimum turning radius and *r*_*k*_(∙) = *r*_*k*_ − *p*_*min*_. *o* is the simply connected area of obstacle model in obstacle map. When there are many obstacles, o will increase the subscript. For example, *o*_*l,l=1,…,n*_*.* *ψ* is an obstacle model. *δ* (*s*_*k*_*,* *ψ*) is the distance between the pose of the mobile robot and the obstacle model. The minimum distance of all obstacles is constrained by inequality *δ*_*min*_:

19 $$ o_{k} (s_{k} ) = [\delta (s_{k} ,\psi_{1} ),\delta (s_{k} ,\psi_{2} ), \ldots ,\delta (s_{k} ,\psi_{R} )]^{T} - [\delta_{\min } ,\delta_{\min } , \ldots ,\delta_{\min } ]^{T} $$

where *v*_*k*_ is the linear velocity of the robot at the pose *s*_*k*_. *v*_*k*_ is defined as:

20 $$ v_{k} = \frac{{p_{k} \Delta \theta_{k} }}{{\Delta T_{k} }}\gamma (s_{k} ,s_{k + 1} )\mathop \approx \limits^{{\vartriangle \theta_{k} \ll 1}} \frac{{\left\| {d_{k} } \right\|_{2} }}{{\Delta T_{k} }}\gamma (s_{k} ,s_{k + 1} ) $$

where *γ*(∙) ∈ [−1,1]is sign of mobile robot velocity. The result of mapping the direction vector *q*_*k*_ = [ *cosθ*_*k*_, *sinθ*_*k*_, 0] ^T^ to the distance vector *d*_*k*_ is:

21 $$ \gamma (s_{k} ,s_{k + 1} ) = sign(\left\langle {q_{k} ,d_{k} } \right\rangle ) \approx \frac{{\kappa \left\langle {q_{k} ,d_{k} } \right\rangle }}{{1 + \left| {\kappa \left\langle {q_{k} ,d_{k} } \right\rangle } \right|}} $$

where < .,. > is the scalar computor. The angular velocity is *w*_*k*_ = *∆θ*_*k*_*/∆T*_*k*_ and |*w*_*k*_|≤ *w*_*max*_ the conditions are satisfied. *w*_*max*_ = *v*_*max*_*p*^−1^_ *min*_. Linear acceleration is described as:

22 $$ a_{k} = \frac{{2(v_{k + 1} - v_{k} )}}{{\Delta T_{k} + \Delta T_{k + 1} }} $$

where linear velocity and angular velocity constraints are *v*_*k*_(*s*_*k*+*1*_*,s*_*k*_*,∆T*_*k*_) = [*v*_*max*_ − *|v*_*k*_*|,* *w*_*max*_ − *|w*_*k*_*|*]^T^, the inequality is *a*_*k*_ (*s*_*k*+*2*_, *s*_*k*+*1*_, *∆T*_*k*+*1*_, *∆T*_*k*_) = *a*_*max*_ − *|a*_*k*_*|*. The precise nonlinear programming is transformed into an approximate nonlinear square optimization problem. The optimization of the solution formula is achieved through the use of the approximate least squares method. The solver's properties are harnessed to approximate the first derivative, ensuring an effective solution. The equality constraint, denoted as *h*, is represented by the quadratic penalty with a scalar weight *σ*:

23 $$ \phi (h_{k} ,\sigma_{h} ) = \sigma_{h} h_{k}^{T} Ih_{k} = \sigma_{h} \left\| {h_{k} } \right\|_{2}^{2} $$

The inequality approximates the weighted unilateral quadratic penalty:

24 $$ \chi (v_{k} ,\sigma_{v} ) = \sigma_{v} \left\| {\min \left\{ {0,v_{k} } \right\}} \right\|_{2}^{2} $$

Equation (24) can be approximated by the overall unconstrained optimization of the objective function $\mathop V\limits^{\sim } (B)$ :

25 $$ B^{*} = \arg \mathop {\min }\limits_{{B\backslash \left\{ {s_{1} ,s_{n} } \right\}}} \mathop V\limits^{\sim } (B) $$

26 $$ \mathop V\limits^{\sim } (B) = \sum\limits_{k = 1}^{n - 1} {[\Delta T_{k}^{2} + \phi (h_{k} ,\sigma_{h} ) + \chi (\mathop {r_{k} ,}\limits^{\sim } \sigma_{r} ) + \chi (\upsilon_{k} ,\sigma_{\upsilon } )} + \chi (o_{k} ,\sigma_{o} ) + \chi (\alpha_{k} ,\sigma_{\alpha } )]{ + }\chi (\alpha_{n} ,\sigma_{\alpha } ) $$

where B* is the optimal solution vector. When ownership values all approach infinity, B* coincides with the actual minimum value of Eq. (25). In order to solve (Eq. 12), TEB algorithm adopts Levenberg Marquardt (LM) method. The graph optimization framework g2o implements an efficient sparse variant of LM.

### Algorithm fusion

**Figure 7:**
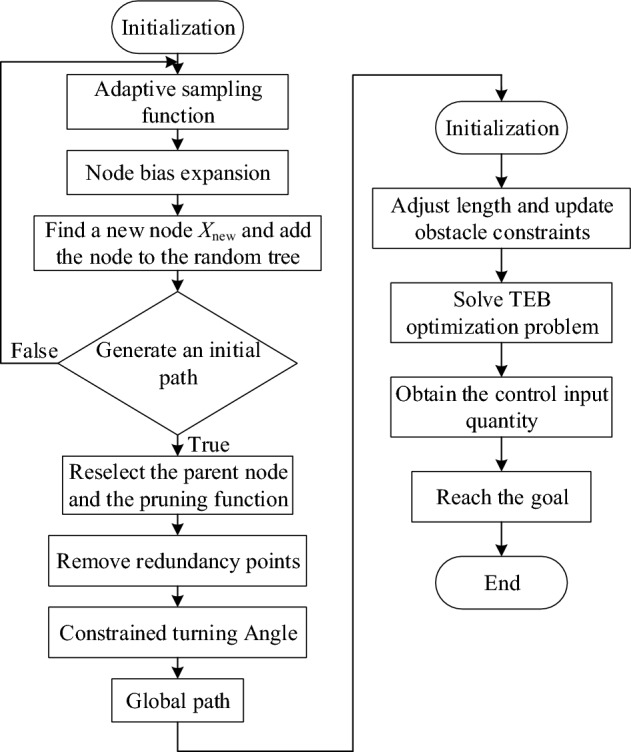
Fusion algorithm flow chart.

Combining the global and local path planning algorithms creates a hybrid algorithm. The global path planning involves multiple steps: firstly, employing an adaptive sampling function to generate random sampling points; secondly, generating a new node through node bias and adding it to the random tree; thirdly, removing redundant points in the initial path; fourthly, imposing constraints on the path turning angle and smoothing the path. The resultant global path information is then fed into the local path planning algorithm. The local path planning process consists of obtaining the path, applying kinematics constraints to the trajectory, verifying the trajectory, finally calculating the control inputs. This iterative process continues until the mobile robot reaches the target point, as depicted in Fig. 7.

## Experimental

To evaluate the feasibility and effectiveness of the enhanced algorithm described in this paper, the Robot Operating System (ROS) serves as the operational environment. The simulation experiment takes place on a computer, and the prototype experiment is implemented on the mobile robot, enabling a thorough assessment.

**Figure 8:**
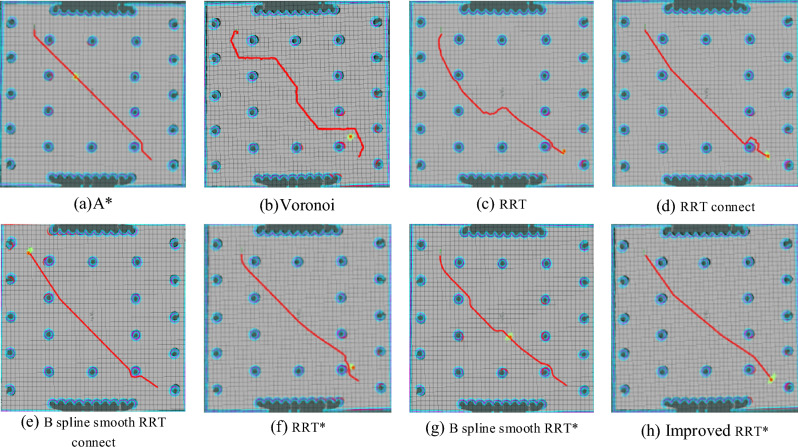
Algorithm comparison diagram.

**Figure 9:**
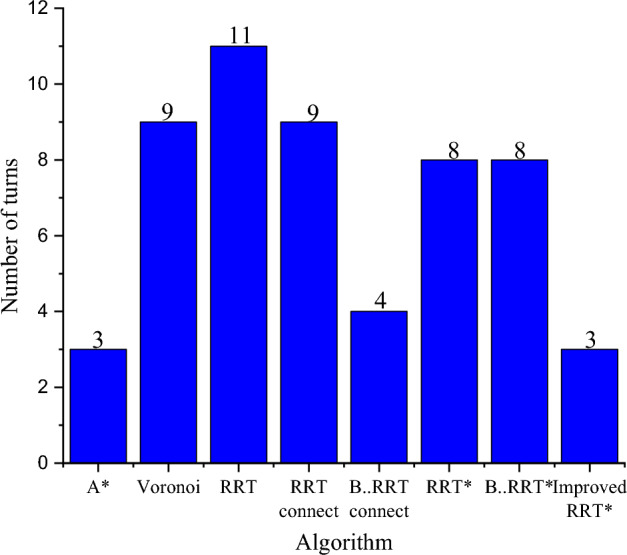
Number of turns diagram.

The model is constructed using Gazebo, a sophisticated robot simulation software renowned for its high-fidelity physical simulation capabilities. Gazebo excels in accurately and efficiently replicating the intricate operations of robots within diverse indoor and outdoor settings. As a simulator, it swiftly validates the algorithm's effectiveness by creating a Gazebo model based on the experimental map. Following the construction of the simulation model, the next step involves employing the gmapping technique. This step is crucial for validating the improved RRT* algorithm's effectiveness. To thoroughly assess its performance, the A* algorithm, Voronoi algorithm, RRT algorithm, RRT connect algorithm, B spline smooth RRT connect algorithm, RRT* algorithm, B spline smooth RRT* algorithm, and the enhanced RRT* algorithm are individually executed within this mapped environment. The trajectory, depicted in Fig. 8, delineates specific elements: the gray area signifies safety, while the black cylinder represents obstacles, and the blue border denotes obstacle expansion. The mobile robot is denoted by the orange dot, and the red path illustrates the route planned by the global algorithm. Corresponding data is presented in Table 1 and Fig. 9. Notably, the path generated by the improved RRT* algorithm, as demonstrated in Fig. 8, exhibits a smoother trajectory compared to traditional algorithms. It significantly reduces the number of turning points, effectively enhancing the overall path quality. Further analysis, as evidenced in Table 1 and Fig. 9, reveals that compared to the traditional RRT* algorithm, the improved version reduces the path length by 5.8% and decreases the number of turning points by 62.5%. This reduction in path length enables the mobile robot to reach its destination more swiftly, while fewer waypoints conserve the robot's memory usage.

**Table 1:** Comparison of algorithm performance indexes before and after improvement.

Algorithm	Path length(m)	Number of nodes	Time to plan a path (s)
A*	20.21	291	384.93
Voronoi	27.20	461	97.09
RRT	22.53	173	70.99
RRT connect	23.11	159	61.26
B spline smooth RRT connect	21.43	963	158.07
RRT*	20.72	86	49.10
B spline smooth RRT*	22.97	749	76.1
Improved RRT*	19.52	771	52.10

**Figure 10:**
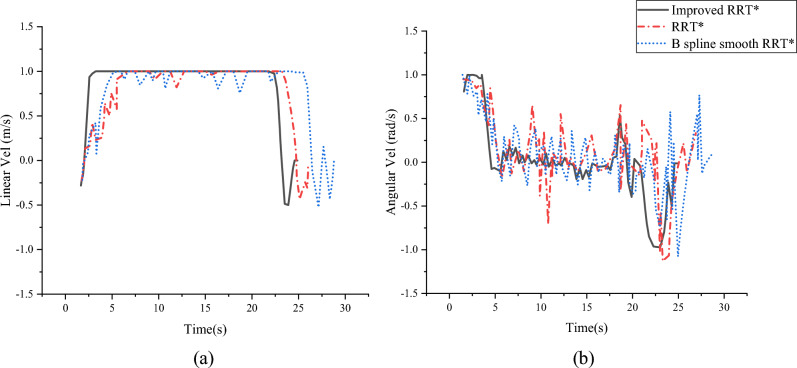
Simulation model velocity diagram.

**Figure 11:**
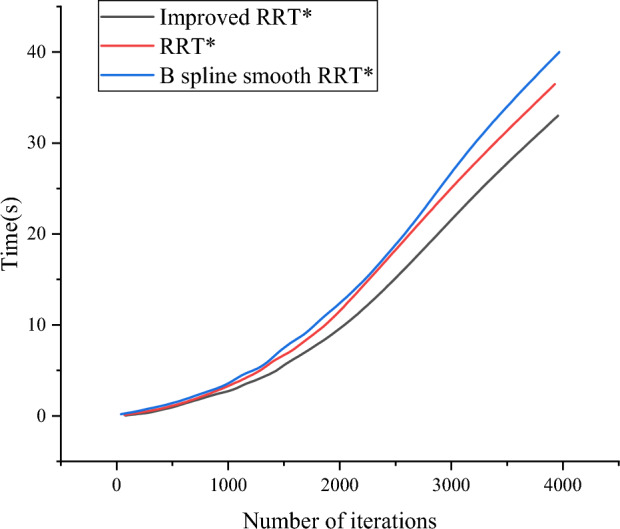
Time and number of iterations.

The resulting trajectory aligns with expectations. As illustrated in Fig. 10, The angular velocity and linear velocity of various RRT* algorithms applied to mobile robots are obtained and compared. In Fig. 10, the linear velocity consistently remains within the range of [−0.5 m/s, 1 m/s], The angular velocity also remains within the range of [-1 rad/s, 1 rad/s]. Of particular note is that only the improved algorithm maintains a constant speed of 1 m/s between [5 s, 20 s], indicating a smooth operation of the mobile robot during this time interval. Compared with other algorithms, the angular velocity fluctuation range of the improved algorithm is the smallest in the period of [5 s,18 s]. These results confirm that the improved algorithm is more consistent with the kinematic model of the mobile robot, and can follow the planned path more effectively and move more smoothly. As can be seen from Fig. 11, in the environment of Fig. 8, the optimization convergence speed of the three algorithms is relatively close, but the Improved RRT^*^ algorithm has the best curve, which can effectively improve the efficiency of path planning and reduce the time of path planning.

**Figure 12:**
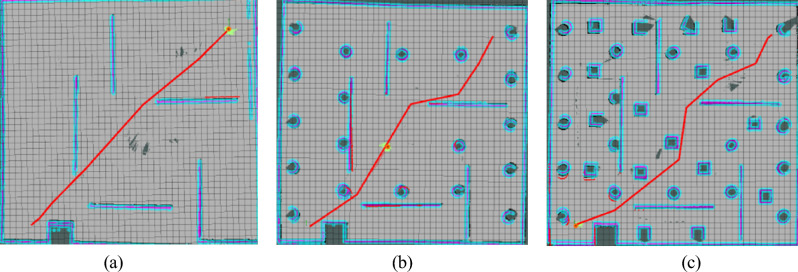
Algorithm simulation diagram under multiple maps.

**Figure 13:**
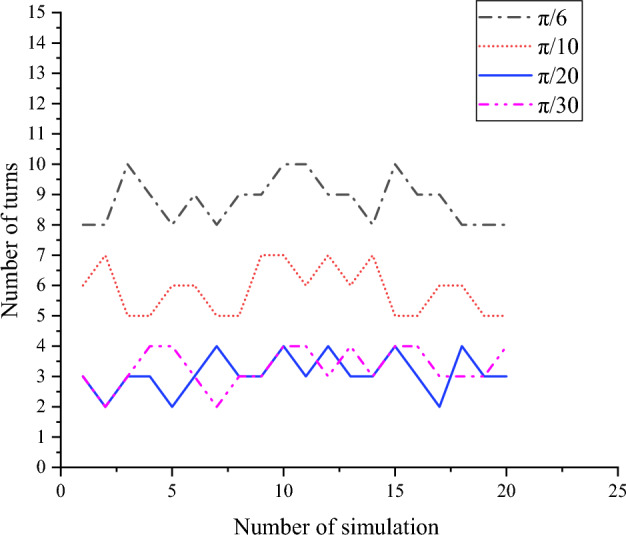
Influence of angle deviation threshold on improved RRT* algorithm.

**Figure 14:**
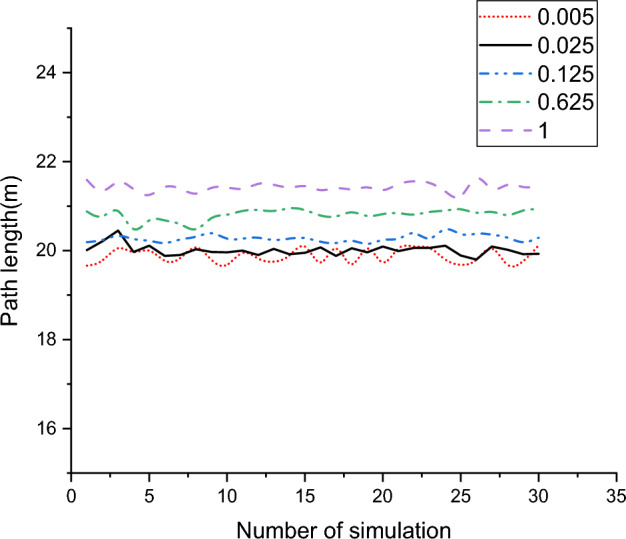
Influence of waypoint distance on improved RRT* algorithm.

As can be seen from Fig. 12, the Improved RRT^*^ is put into the map of three different environments for simulation experiments, and the Improved RRT^*^ successfully generates the optimal path without colliding with obstacles. The results show that the Improved RRT^*^ is relatively robust and can successfully plan paths and avoid obstacles in different environments. In Fig. 13, the influence of Angle deviation threshold *A*_*d*_ on the number of path turns is analyzed, and it is found that setting the Angle at about π/20 degrees generates fewer path turns and smoother path. In Fig. 14, the relationship between the distance between the waypoints and the path length in the improved RRT^*^algorithm is analyzed. As shown in the figure, when the distance between the waypoints reaches 0.025 m, the impact on the distance is small, so 0.025 m is selected as the path point distance of the algorithm.

**Figure 15:**
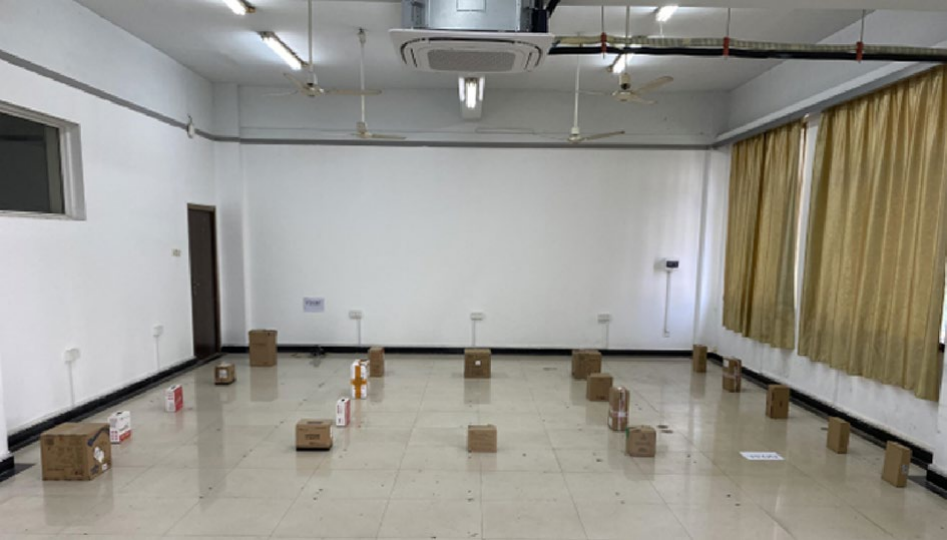
Experiment environment diagram.

**Figure 16:**
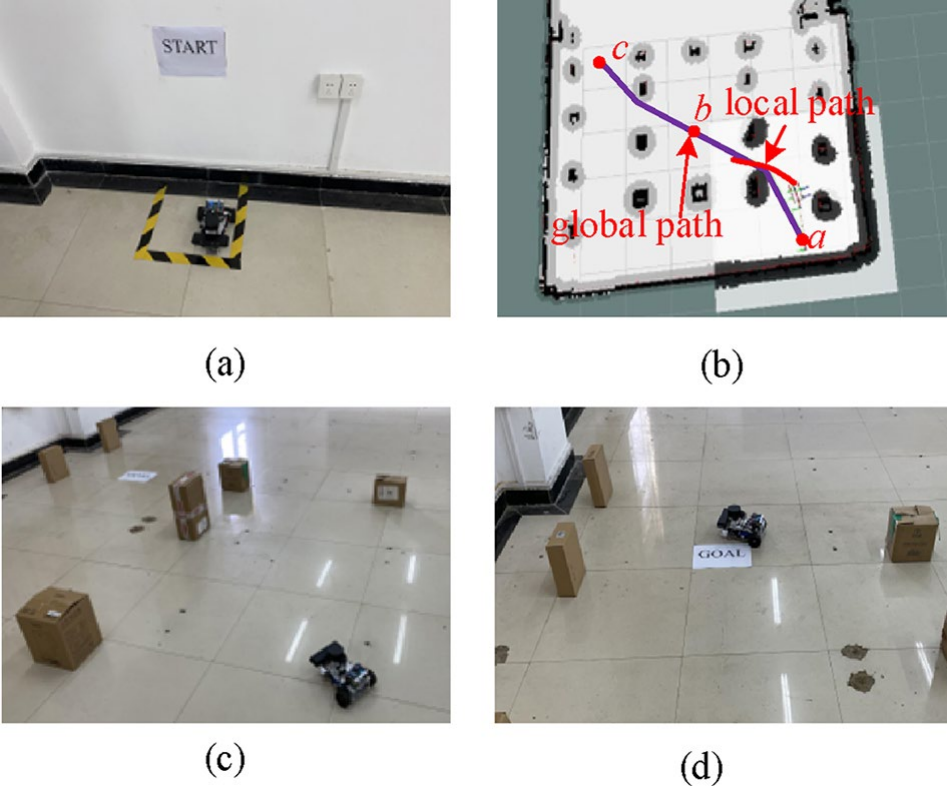
Mobile robot experiment diagram.

The hybrid algorithm is implemented in the mobile robot, and its effectiveness is verified through experimentation in a controlled environment, as illustrated in Fig. 15. Within the constructed environment, a hybrid algorithm is employed for path planning, and the outcome is depicted in Fig. 16. In Fig. 16b, point a designates the initial position of the mobile robot, point b represents the intermediate node during the robot's movement, and point c signifies the ultimate goal of the mobile robot. The purple path illustrates the global trajectory generated by the enhanced RRT* algorithm, while the red path depicts the local trajectory produced by the optimized TEB algorithm. Using this algorithm for path planning guarantees a smooth trajectory that matches the robot's kinematic traits. Crucially, it avoids collisions with obstacles, achieving the intended result.

## Conclusion

Within this paper, introduce a fusion algorithm that synergizes the advancements of the improved RRT* algorithm and the TEB algorithm. The proposed approach not only expedites the generation of an optimal global path but also refines the trajectory, taking into careful consideration the dynamic constraints inherent to the mobile robot. The global path planning algorithm commences by employing an adaptive sampling function. Subsequently, Afterward, nodes expand with bias, redundant points are removed from the path, and turning angles are constrained. Perform kinematic modeling for the mobile robot. Incorporate dynamic constraints specific to the mobile robot into the local path planning algorithm, effectively integrating the algorithms. This holistic approach empowers the algorithm to strategize the mobile robot's path, leading to the attainment of a path of high quality.

In forthcoming research efforts, the emphasis transitions to utilizing a fusion algorithm for path planning within the framework of multiple robots. This necessitates addressing concerns such as priority assignment and potential conflicts in paths among the robots. The collaboration of multiple robots holds the promise of extending the operational radius and enhancing overall efficiency. Concurrently, there is an examination of autonomously assigning execution tasks and areas to mobile robots. This strategy is designed to enhance robot efficiency and markedly improve the quality of work. Furthermore, the research delves into investigating how mobile robots navigate and avoid dynamic obstacles.

## Data Availability

The datasets used and analysed during the current study available from the corresponding author on reasonable request.
